# "We don’t treat your kind": Assessing HIV health needs holistically among transgender people in Jackson, Mississippi

**DOI:** 10.1371/journal.pone.0202389

**Published:** 2018-11-01

**Authors:** Amaya Perez-Brumer, Amy Nunn, Elaine Hsiang, Catherine Oldenburg, Melverta Bender, Laura Beauchamps, Leandro Mena, Sarah MacCarthy

**Affiliations:** 1 Department of Sociomedical Sciences, Columbia University, New York, New York, United States of America; 2 Department of Behavioral and Social Sciences and the Rhode Island Public Health Institute, Brown University School of Public Health, Providence, Rhode Island, United States of America; 3 School of Medicine, University of California, San Francisco, California, United States of America; 4 Francis I Proctor Foundation, University of California, San Francisco, California, United States of America; 5 Department of Ophthalmology, University of California, San Francisco, California, United States of America; 6 Department of Epidemiology & Biostatistics, University of California, San Francisco, California, United States of America; 7 Division of Infectious Diseases, University of Mississippi Medical Center, Jackson, Mississippi, United States of America; 8 Department of Population Health Science, John D. Bower School of Population Health, Jackson, Mississippi, United States of America; 9 Rand Corporation, Santa Monica, California, United States of America; University of California Los Angeles, UNITED STATES

## Abstract

HIV disproportionately impacts transgender communities and the majority of new infections occur in the Southern United States. Yet, limited data exists on contextual realities of HIV vulnerability and healthcare needs among transgender individuals in the Deep South. Addressing this gap in the literature, we assess the health needs, including barriers and facilitators to accessing healthcare, including and beyond HIV, from the perspective of transgender men and women in Mississippi. Between June-August 2014, in-depth, semi-structured qualitative interviews (n = 14) were conducted with adult transgender persons at an LGBT healthcare setting in Jackson, Mississippi. In-depth interviews lasted between 60–90 minutes and followed semi-structured format (themes probed: HIV vulnerability, healthcare needs, and availability of gender-affirming medical care). Audio files were transcribed verbatim and analyzed using Dedoose (v.6.1.18). Among participants (mean age = 23.3 years, standard deviation = 4.98), 43% identified as a transgender man or on a transmasculine spectrum, 43% as Black, and 21% self-reported living with HIV. HIV-related services were frequently described as the primary gateway to accessing healthcare needs. Nonetheless, participants’ primary health concerns were: gender affirmation processes (hormones, silicone, binding/packing); mental health; and drug/alcohol use. Stigma and discrimination were commonly reported in healthcare settings and health-related information was primarily attained through social networks and online resources. Results highlight gender identity alongside race and pervasive marginalization as key social determinants of transgender health in Mississippi. As Mississippi is one of several states actively debating transgender access to public accommodations, findings underscore the need to treat transgender health as a holistic and multidimensional construct, including, but moving beyond, HIV prevention and care.

## Introduction

Increasing transgender visibility has drawn attention to the range of health disparities and unique public health needs faced by transgender individuals across the United States. Available literature reports that close to 1 million people in the US self-identify as transgender, approximately 0.3% of adults. [1] Transgender individuals are disproportionately burdened by HIV and AIDS, alcohol, tobacco, and drug abuse, obesity, cardiovascular disease, and certain cancers. [2–6] Additionally, high prevalence of mental health problems, including depression, anxiety, and suicidal ideation and attempts, have been documented and are associated with discrimination and victimization. [7–10]

To date the emerging literature on transgender health has been largely focused on HIV due its heightened impact among transgender women. As evidenced, the prevalence of HIV among transgender women in the US is 21.7% (95% CI 18.4–25.1%). [11] and has been estimated to be as high as 24.9% among Black transgender women. [10] In addition the epidemic is disproportionately clustered among key geographic regions. The Southern US accounts for 37.1% of the American population, but 57.6% of all individuals newly diagnosed with HIV and 37.6% of all individuals newly diagnosed with AIDS cases reside in the South. [12] Nonetheless, an understanding of the factors driving HIV disparities is limited among transgender individuals in the South. Public health efforts are lacking, and Mississippi, in particular, is experiencing an HIV and AIDS crisis as the state with the highest HIV incidence and AIDS mortality in the nation. [13]

Nonetheless, the focus on HIV has also had the consequence of overshadowing the comprehensive health needs of transgender individuals. For example, a range of transgender-specific barriers, from lack of provider knowledge and competency to provider rejection, violence, and discrimination, further compounds these health disparities. [14–17] Importantly, one-fifth of respondents in the National Transgender Discrimination Survey—the only nationally representative data on transgender health available–reported denial of care due to their gender identity, with higher numbers among transgender people of color. [10] These experiences drive many individuals to seek care outside legal medical professions, [15,16] or to avoid seeking care altogether. [10] Further understanding the mechanisms through which stigma and discrimination function to limit access to care for transgender individuals is particularly important in the context of federal programs such as the Affordable Care Act (ACA). [18,19] Under Section 1557 of the ACA, federal protections prohibit discrimination on the basis of sex for any healthcare entity receiving federal funds, and, in 2016, a formal clarification was made to include anti-transgender discrimination. However, private insurers not receiving federal funds are not subject to the ruling, and current controversy regarding a possible rollback of Section 1557 makes the coverage of transgender individuals and transition-related care vulnerable to misinterpretation and discontinuation. [18]

The majority of literature exploring health needs of transgender people has focused on metropolitan settings in states that have historically enforced transgender-protective policies [20] (i.e., Democratic leaning) and the health needs of transgender people in the Deep South are relatively unknown. [21,22] As a result, limited scholarship has assessed the relationship between structural stigma, defined as the societal-level conditions including institutional policies and cultural norms that constrain opportunities, resources, and well-being, [9] and heath outcome for transgender individuals in the Deep South. The structural stigma experienced by transgender individuals in the South remains substantial and exacerbated by legislation that ignores trans-inclusive Title IX guidelines and limits birth certificate changes. [23] This is particularly relevant today as evidenced by the most recent wave of “bathroom bills,” whereby states such as North Carolina, South Carolina, and Oklahoma have passed or introduced laws requiring public bathrooms to be segregated by sex assigned at birth. Especially in Mississippi, where recent legislation (i.e., House Bill 1523) [24] further restrict access to critical health services to gender and sexual minority patients, research is urgently needed to assess transgender health needs.

The awareness of transgender health in public health underscores significant advancements in understanding the associations between gender identity and health disparities. However, limited research to-date has taken into account the spectrum of health-related needs, including and extending beyond HIV. Further attention must be paid to the health and service needs of transgender individuals residing in the Deep South (referring to a subregion in the Southern United States including Alabama, Georgia, South Carolina, Mississippi, and Louisiana). Addressing this gap, this study sought to provide a qualitative portrait of the health needs from the perspective of transgender men and women in Mississippi by assessing: (1) healthcare needs and (2) barriers and facilitators to accessing healthcare.

## Methods

Between June-August 2014, in-depth qualitative interviews (N = 14) were conducted with adult transgender persons at an LGBT healthcare setting in Jackson, Mississippi. The Institutional Review Board (IRB) from the Miriam Hospital at Brown University in Providence, Rhode Island and University of Mississippi Medical Center in Jackson, Mississippi reviewed and approved all study procedures.

### Data collection procedures

In efforts to maximize the variety of perspectives obtained in sampling, a purposive sampling approach [25] was implemented that actively recruited people from the transgender community who identified on a transfemine and transmasculine spectrum, as well as, people who self-described their race as Black or White. Recruitment was conducted with clinical staff and potential participants were identified through the course of routine clinical care. Potential participants were informed that this study consisted of a one-time anonymous qualitative interview seeking to better understand healthcare needs of the transgender community in Mississippi and barriers and facilitators to accessing healthcare. Research procedures were described orally by study staff and written informed consent to participate was obtained at the beginning of each interview. Participants were compensated $30 for their time.

Interviews lasted 60–90 minutes and were conducted in a private and confidential room at the LGBT healthcare center which participants were familiar with. All interviews were audio recorded, and followed a semi-structured interview guide addressing topics related to: gender identity, access medical services, experiences with providers, health needs, and facilitators to healthcare. Wording of interview questions were adjusted to accommodate the pronouns of choice identified by participants, to engage in open-ended discussion, and follow-up probes. All interviews were conducted by Perez-Brumer, a Latina cisgender female researcher, who holds an MSc and is currently a PhD candidate. She is trained in qualitative methodology, public health, gender and women’s studies, and social epidemiology and has almost ten years of experience conducting research with transgender communities.

While the analytic sample size from which these results are derived are from 14 in-depth qualitative interviews, 6 formative interviews (which were not included in this analysis) where conducted with community leaders to modify and adapt final semi-structured interview guide utilized in interviews to this specific context. Given the preliminary nature of the research questions, which aimed to broadly assess the healthcare needs and barriers and facilitators to healthcare among transgender people in Mississippi, interviews were conducted until a saturation was achieved. [26,27] Notably, due to the scant available research in this context, these findings serve as a first step to inform scholars and public health practitioners of the health needs and more in-depth mixed-methods follow-up assessments are encouraged.

### Analytical approach

Interview audio recordings were transcribed verbatim. Transcripts were analyzed using an immersion crystallization approach to identify themes and relationships between themes. [28] Initial set of themes was transformed into codes and coding was carried out using Dedoose v.6.1.18, (2014, Los Angeles, CA: SocioCultural Research Consultants, LLC, www.dedoose.com). Three members of the study team created a codebook which included codes, brief descriptions, and illustrative quotations. Prior to coding, coders completed training sessions to practice applying the final set of codes to a subset of excerpts. To finalize the codebook and analytic plan, the constant comparison method was used to compare and re-review coded transcript data to ensure that coded data were as representative as possible.

## Results

Among participants (mean age = 23.3 years, standard deviation = 4.98), slightly fewer than half (43%) identified as a transgender man or on a transmasculine spectrum, and 43% self-reported their race as Black. While HIV serostatus was not explicitly asked of participants, 21% discussed living with HIV. See Table 1 for demographic characteristics. Results from thematic analysis of qualitative interviews are grouped into three major emergent themes: (1) Health needs beyond HIV; (2) Barriers to engagement in routine medical care; and (3) Economic, social marginalization, and health vulnerabilities: (1) Health needs beyond HIV; (2) Barriers to engagement in routine medical care; and (3) Economic, social marginalization, and health vulnerabilities.

**Table 1:** Demographic characteristics of transgender study participants in Jackson, MS.

Characteristic	Gender identity^a^		
	*n (percent*^b^*)*		
	All (n = 14)	Transfeminine spectrum (n = 8)	Transmasculine spectrum (n = 6)
Age, years^c^			
18–24	9 (64)	5 (63)	4 (67)
25–30	4 (29)	2 (25)	2 (33)
31–37	1 (7)	1 (13)	0 (0)
Race/ethnicity			
Black	6 (43)	4 (50)	2 (33)
White	7 (50)	3 (38)	4 (67)
Multiracial	1 (7)	1 (13)	0 (0)
Employed	9 (64)	4 (50)	5 (83)
Health insurance^d^	5 (36)	1 (13)	4 (67)
Living with HIV	3 (21)	3 (38)	0 (0)

^a^ Self-reported gender identity as on the transfeminine spectrum (MTF, male-to-female) or transmasculine spectrum (FTM, female-to-male).

^b^ Percent of sample or subsample. Total percentages may exceed 100 due to rounding error.

^c^ The mean age was 23.3 years, with a standard deviation of 4.98.

^d^ Insured status includes coverage via Medicaid.

### Health needs beyond HIV

Across interviews the theme that HIV services were perceived to dominate and minimize other healthcare needs among the transgender community emerged. For example, one participate stated: *“I guess you could call it LGBT health*, *you know*, *[but] it’s all kind of associated with HIV*, *which is kind of crazy” [Transmasculine*, *White*, *25 years old]*.

While services specifically for those with HIV were voiced as an important and needed resource, there were also tensions associated with restricting the eligibility of services based on HIV status. One participant described: “*[A certain clinic is] for patients only who have HIV so… I couldn’t go there” [Transmasculine*, *White*, *20 years old]*. Across interview narratives, vulnerability to HIV was perceived as more relevant to transgender women compared to transgender men: “*I think HIV plays a big part in the trans community when it comes to trans women” [Transmasculine*, *Black*, *25 years old]*.

The association between HIV and being transgender was commonly underscored as a recurrent source of stigma encountered in healthcare settings and often resulted in medical mistrust. When discussing a recent hospitalization due to pneumonia, one participant expressed discomfort and anger over the assumption that they were living with HIV: *“after they found out I was trans*, *they treated me like… an HIV-ridden gay male and told me that my pneumonia was not pneumonia*, *it was that I had HIV*, *which I don’t have*. *That was very*, *very upsetting… [they] find out that I’m trans and then instead of just focusing on the actual issue… it’s suddenly all different because you’re a deviant” [Transfeminine*, *White*, *24 years old]*.

When explicitly asked about the top three healthcare needs for the transgender community in Mississippi, participants most commonly identified access to safe and legal cross-sex hormones (27.0%), transgender health trained providers (18.9%), and mental health services (16.2%).

### Barriers to engagement in routine medical care

A second emergent theme were that barriers to engagement in routine medical routine were deeply intertwined with pervasive stigma and discrimination within clinical settings. As illustrated: *“I feel like transgender people in the medical community are almost looked at as an “it”*, *instead of a normal patient” [Transmasculine*, *White*, *20 years old]*.

Narratives highlighted negative interactions with receptionists, judgmental stares in the waiting rooms, and rejection and disgust from providers during scheduled visits. When calling to inquire about endocrinology services, one participant recounted:

“*I called around… and I said do any of your doctors do HRT [hormone replacement therapy]*? *And they’re like*, *yeah*, *do you need estrogen*? *Do you have problems with that*? *And I was like*, *no ma’am*, *I don’t*. *I was like*, *I need testosterone*. *And she was like*, *what*? *I said I am a transgender patient and she said*, *oh*, *we just don’t do that*, *click and hung up”*

*[Transmasculine, White, 19 years old*].

Transphobia expressed by providers and clinic staff was highlighted as a barrier to finding medical services willing to treat transgender patients. For example: *“I’ve been turned down by five different doctors before I came here… because I wanted to start my transition” [Transfeminine*, *White*, *37 years old]*. Another participant explained that after years of care from their family physician, the rejection of a trusted medical provider was deeply felt: “*That hurt*. *I cried for days over that [rejection] because he was the one I really wanted to take care of me because I really trusted him… but he said he didn’t believe in it [being transgender]*, *that he would not do it” [Transfeminine*, *Black*, *27 years old]*. Furthermore, in seeking top surgery, one individual emphasized the consistent presence of discrimination based on gender identity and outrage due to transphobic experiences with medical providers:

“…*surgeons out here are cruel*, *when it comes to trans*. *[They say] we don’t do implants on men*. *I’m saying to myself like that’s so rude*. *Like I’m clearly a transgender person*. *I live my life as a woman*. *How dare you*?*”*

*[Transfeminine, Black, 25 years old*].

Lack of provider knowledge related to transgender-specific health needs often drove provider stigma and discrimination. For example:

“*I go in and say hey, I need a Pap, and I look like this [masculine physical presentation], they’re gonna say… what? You need to get your prostate checked? No, I need a Pap. Prostate? Pap. Then they’re gonna be asking questions, well why do you need that? And… so when you have somewhere you don’t have to go through all the questions and interrogations and feeling uncomfortable… ‘cause after awhile you’re gonna be like, okay, you know what? Just forget it. I’ll just deal with it on my own*”

*[Transmasculine*, *Black*, *27 years old*].

Participants reported a sense of demoralization accompanying their experiences: *“discrimination and stigma… [is] like getting punched in the gut by a really*, *really strong*, *like*, *bodybuilder or something*, *like it knocks the wind out of you and like you have to sit there and think about that all day” [Transmasculine*, *White*, *19 years old]*. This led many to use informal avenues of care, such as black markets, for hormones or injectable silicone. One participant summarized these sentiments of exasperation with formal medical care:

“*…when you have gender dysphoria*, *like when you just really cannot stand yourself*, *you’re desperate*, *because you know you don’t have them in your resources*, *then you’ll take whatever you can whether it’s life-threatening or not… I’ll just go ahead and do it and I’m sure that’s how other trans-people think”*

*[Transfeminine, Black, 27 years old*].

### Economic, social marginalization, and health vulnerabilities

Narratives also highlighted the critical intersection between economic and social marginalization, health vulnerabilities, and limited access to health care. Economic marginalization was primarily referenced when discussing lack of employment opportunities, especially when transitioning, contributing to the high prevalence of drug use and engagement in survival sex work: *“A lot of us become homeless*, *don’t have anywhere to go so we turn to drugs*. *We turn to sex*. *We start being careless*, *start slipping up” [Transfeminine*, *Black*, *25 years old]*. Given the dearth of economic opportunities several participants described the frequency of sex work. For example, *“Let me tell you something*, *it’s not even about being transgender*, *a lot of people prostitute because they don’t got money” [Transmasculine*, *White 19 years old]*. Yet, despite the reality of limited employment opportunities, both poverty and sex work were stigmatized within the community:

“*You’re going to find a lot of people that say*, *oh*, *she’s a coin girl… [and] at the same time those are the same people saying*, *she’s nasty*, *she’s an escort*, *she sells to everybody*, *she’s a prostitute*. *People*, *they don’t want to see you just sleeping on people’s floors*, *not just being broke*, *but at the same time they don’t want to see you prostitute”*

*[Transfeminine, Black, 22 years old*].

Dimensions of social marginalization were also described as interwoven with sexual risk behavior. For some participants, sexual risk taking, such as, engaging in sex with multiple partners was described in part as an external form of seeking affirmation of their sexuality and attractiveness. For example:

“*I’ve learned now that I was just trying to feel feminine*, *I guess*. *I thought that if tons of men wanted to be with me that meant that I was succeeding in what I wanted*. *I was feminine*, *and pretty*, *and wanted*, *you know*, *by somebody… so that’s why I did it at first and I was sleeping with multiple people… I think… well*, *I slept with a few people and thank God I didn’t catch anything”*

*[Transfeminine, White, 24 years old*].

For other interviewees, while sexual attraction was a component, additional factors, such as, employment needs and economic motivations further influenced sexual risk including limited condom use.

“*Okay, the three guys I had been with before him [boyfriend] didn’t wanna use a condom, so, um, which may be dumb but I was okay with them not using a condom. They were hot and they said they were clean. I trusted that. I’m clean. Um, so I trust them. I mean I would want guys to use a condom but it’s like I’m not gonna fight about it especially if they are paying*”

*[Transfeminine, Black, 18 years old*].

Importantly, these experiences with economic and social marginalization were described in relation to race. Racism, described by both White and Black participants, emerged as a crucial modifier to existing health disparities. As described, *“Yeah it’s hard for Blacks in Mississippi*, *it’s even harder when you are trans” [Transmasculine*, *White*, *20 years old]*. Another participant noted:

“*It’s always been harder on Blacks in general ‘cause we’re minority and it’s always been a struggle, but then the fact that you’re Black and you’re trans is a double standard… because people don’t just want us to succeed and then, you know, I think this is why… pardon me, I’m having so much of a problem in the education part of it or whatever, because there are not that many trans-women doing what I’m doing, going… down here in the south that is, going through school, trying to get my education as anyone else would*”

*[Transfeminine, Black, 27 years old*].

Additionally, race was discussed as a factor that divided resources and community openness from within the LGBT community in Jackson. As described:

“*We have some straight/gay alliances and stuff for the LGBT community here in Jackson…we have a parade, but even that is segregated because it’s more for the Caucasian people that have the parade and it’s right over there in that area. And that bothers me because you go to other places and they have whole gay districts, they have huge, national parades. I know so many people in the LGBT community here that goes to Memphis Pride and Atlanta Pride, and here when we have Pride it’s not for the whole community*”

*[Transfeminine, Black, 22 years old*].

## Discussion

Our study furthers the existing literature on health needs of the transgender community by including diverse perspectives from transgender Mississippians, a population which to date has not been scantly represented in public health scholarship. While epidemiological trends underscoring the disproportionate burden of HIV on transgender women and other health disparities in the Deep South cannot be ignored, [29,30] it is important to conceptualize transgender health (across both transmasculine and transfeminine needs) as a multidimensional construct within which HIV is one aspect. While the most pressing healthcare needs described by the transgender community in Mississippi were access to safe and legal cross-sex hormones, transgender health trained providers, and mental health services, HIV was described by many as the only entry point into care. Guided by these findings, we suggest a modified social determinants of health framework that explicitly highlights gender identity as critical to understanding transgender health needs in Jackson, Mississippi.

This work presents a first step in showing how social marginalization and manifestations of transphobia limit willingness to access healthcare, quality of gender-affirming healthcare provided, and increased health vulnerabilities for transgender individuals in Jackson, Mississippi. Participant narratives link to a growing body of literature underscoring the critical role of social determinants of health (SDHs), meaning the structural and social factors that pattern the distribution of conditions and foster health and access to health services. [31] Yet, there is only limited attention to gender identity as a SDH, [32] and these results suggest that gender nonconformity functions as an additional social factor compounding inequalities in health outcomes and access to health resources. Examples of social determinants of health include occupation, education, income, gender, and race/ethnicity, [31] and for transgender and gender nonconforming individuals, discrimination due to their gender identity may further magnifies barriers to care and existing health disparities. [14,16,33] Building on these findings, public health strategies seeking to ameliorate transgender health inequities should understand gender identity as a factor alongside other sociopolitical factors jointly driving adverse health outcomes, such as, housing and economic instability and pervasive experiences with discrimination.

The importance of gender-affirming care, [15,34,35] meaning the interpersonal process by which an individual’s gender identity and expression are socially recognized, was an emergent theme across our data. Participants noted the importance of social recognition (e.g. pronoun usage), and need for medical care awareness and willingness to serve the needs of the transgender community in Mississippi. Furthermore, these results suggest that public health practitioners should look beyond HIV when conceptualizing notions of transgender health to understand SDHs as interactive and cumulative factors. For example, race is a critical factor that cannot be overlooked, both across the US and specifically in the context of the Deep South. Previous studies have demonstrated worse health outcomes for transgender people of color, especially Black transgender women. [22,36,37] Particularly relevant in the context of Mississippi where close to 40% of residents are Black, [38] race and gender identity must be considered as key social factors individually and jointly to assess how multiple cumulative minority statuses impact health. Frameworks such as the theory of intersectionality [39] and its applications to understandings of sexual and gender diversity in the Deep South [40] are may provide utility for work furthering how various social factors intersect to constitute inequality and vulnerability.

The results presented here have several limitations. These data are from a small qualitative study from a transgender sample recruited from an LGBT healthcare clinic in Mississippi, therefore, findings may not be generalizable to all transgender and gender nonconforming people in Mississippi or the Deep South. Scholars seeking to build on this work should assess the health needs of transgender individuals not currently engaged in routine care. Semi-structured interview guides were created with the aim of giving a voice to transgender Mississippians engaged in care, not to assess healthcare advice attained through informal sources (i.e., social networks). As such, given the importance of cross-sex hormones, future studies may want to further investigate pathways of hormone attainment and administration (via physician and non-physician means). Additional scholarship is also needed on positive interactions and experiences with healthcare providers to provide insight as to how to combat transphobia and promote transgender cultural competence within healthcare facilities in the Deep South.

Our study underscores the relevance of gender identity alongside race and pervasive marginalization as key social determinants of transgender health. These findings suggest that medical provider trainings and access to mental health and cross-sex hormones are vital next steps in addressing the health needs of transgender individuals in Mississippi. As Mississippi is one of several states actively debating transgender access to public accommodations, public health approaches must treat transgender health as a holistic and multidimensional construct, including, but moving beyond, HIV prevention and care.

## Supporting information

S1 File In-depth qualitative interview guide included as supporting information. (DOCX)
