# Supplementary material for: "We don’t treat your kind": Assessing HIV health needs holistically among transgender people in Jackson, Mississippi
Source: PLoS One. 2018 Nov 1;13(11):e0202389. doi: 10.1371/journal.pone.0202389 (PMC6211621; doi:10.1371/journal.pone.0202389)
Supplement: S1 File — (DOCX) [file pone.0202389.s001.docx]

As we went over in the consent, all of the information you provide will be kept confidential. Just as a reminder our interview will probably last around an hour, but could be shorter or longer depending on how our conversation progresses. Do you have any questions before we begin? May I start the recording? *[Start recording]*

**Good [afternoon/morning] thank you for participating today**! *I have asked you to meet with me in the hopes of learning more about your as experience as a transgender, gender fluid, and/or gender nonconforming person and themes related to medical care and availability for the LGBT community in Mississippi. Some of the questions I will ask you may not want to answer and that is fine. Remember that your participation is completely voluntary. Also please keep in mind that there are no right or wrong answers, I am interested in anything you can share with me.*

1. To begin, I was hoping you could tell me a bit more about yourself?
   1. *PROBES*- Age? Gender? Sexual orientation? Sexual Role? Occupation? Where do you live? How long have you been in MS? Who do you live with?
2. What does gender mean to you? What, if any, is the difference between sex and gender? What, if any, is the difference between sexual identity, gender orientation and sexual role? How well understood do you think these terms are among the LGBT community, medical service providers and/or the general population?
   1. *PROBES*- How might definitions of these terms vary between the different groups previously mentioned (your community, medical providers, general population)? Could you give me examples of how these terms might be defined differently by group?
3. Could you describe some of the medical services or clinics available for the LGBT persons? Do you know of any services specific for gender nonconforming persons – by this we mean male-to-female or female-to-male transgender individuals? What experiences have you had or heard of regarding medical services?
   1. *PROBES-* Any medical challenges have you faced (if any)? Which hospitals or clinics have you primary attended?
4. How do you think medical professionals (both doctors and nurses) generally in Mississippi understand the health needs of the transgender community?
   1. PROBES- Why or why not? What training specifically do you think would be useful?
5. In regards to mental health, would you define what this term means to you? What services specific to mental health are you aware of?
   1. PROBES- Would you describe examples?
6. Could you describe how frequent substance use (including alcohol use, marijuana, stimulants, etc.) is among your social network? Transgender community? LGB community? Mississippi more generally?
   1. PROBES- Which substances specifically do you think are most used among transgender people? Has a medical provider ever spoken to you about substance use? If so, could you give me an example of the type of question that was asked? If not, has there been a time when you wish a medical provider would have inquired about substance use?
7. Could you describe how, if at all, transgender individuals access body modification services? Please describe the process of seeking out these services.
   1. PROBES- How common are gender affirmation procedures? Which types? What is any is more common between cosmetic surgery and/or hormone injection? On average, how much do you think [fill in with procedure previously mentioned] costs?
8. **Now I would like to switch topics slightly to discuss any experience with incarceration or jail **. Can you tell me about your experience or about the experience of your transgender friends who may have been in jail? *[If participant has no experience or thoughts on incarcerated LGBT individuals make note and move on to next question]*
   1. *PROBES –* How did the guards and other inmates interact with you?
   2. *PROBES-* What services do you think would be most difficult for LGB and T individuals to access while in jail or prison? Could you elaborate a bit more on specific services that you have either hear positive or negative reviews about?
9. Thank you for sharing your thoughts and experiences so far. We’d like to ask about another personal area of your life. Can you tell me about your sexual partners? Where you meet them? How you negotiate sex (with respect to whether or not it is protected, what type of positions etc.)?
10. Building on the experiences you have described *[either personal or by extension]* when you are presented with a challenging situation related to your personal health, who do you turn to for support?
    1. *PROBES-* Currently relationship with medical provider? How would you define community? Familial ties? Large or small support structure? Sexual partners?
11. How, if at all, might social support and relationships either encourage or potentially provide a barrier for gender and sexual minority individuals accessing medical care? How do you think that having either many or few supportive individuals might impact how someone deals with their own health?
12. To conclude, what would you say are the top three health care needs for transgender persons in Mississippi? LGB individuals?
    1. *PROBES-* Are there services outside of health care that would also be helpful like legal services, employment services etc?

We have come to the conclusion of the topics I had prepared to discuss today. Are there any further comments you would like to add? **THANK YOU FOR YOUR TIME!**
